# Complete Genome Characterization of Novel Chinese Porcine Deltacoronavirus Strain SD

**DOI:** 10.1128/genomeA.00930-17

**Published:** 2017-10-05

**Authors:** Can Liu, Xing Zhang, Ziqi Zhang, Rui Chen, Zhigang Zhang, Qinghong Xue

**Affiliations:** a Shaanxi Innolever Biotechnology Co. Ltd., Yangling, Shaanxi, China; b Yangling Biological Research Center of Chinese Agricultural Valley, Yangling, Shaanxi, China; c China Institute of Veterinary Drug Control, Beijing, China

## Abstract

A porcine deltacoronavirus (PDCoV) was isolated and identified from feces of diarrheic piglets in Shandong Province of the Chinese mainland, and the complete genome of the Chinese PDCoV strain, SD, was sequenced and analyzed to further characterize PDCoV circulating in China.

## GENOME ANNOUNCEMENT

Porcine deltacoronavirus (PDCoV) is an enveloped, single-stranded, positive-sense RNA virus belonging to the fourth genus, *Deltacoronavirus*, in the family *Coronaviridae* of the order *Nidovirales* (1, 2). PDCoV was detected mostly in the entire small intestine, especially the jejunum and ileum, where it causes severe enteritis, accompanied by diarrhea and vomiting, in piglets. Since 2013, the prevalent PDCoV virus has been detected in the United States, South Korea, China, Japan, Europe, and other places (3–7).

Since the discovery of PDCoV in the United States, we have conducted a surveillance study to detect the presence of PDCoV in China. Fifty-six fecal and intestinal samples from the Hebei, Henan, Shandong, Guangdong, and Shaanxi provinces were collected and tested. Molecular testing was positive for PDCoV and negative for porcine epidemic diarrhea virus (PEDV), transmissible gastroenteritis virus (TGEV), and porcine rotavirus (PRotaV). Subsequently, the complete genome sequence of PDCoV strain SD was generated using a previously described method (5).

The complete genomic sequence of PDCoV SD is 25,414 nucleotides (nt) in length, excluding the 3′ poly(A) tail. The genomic organization of this Chinese PDCoV is the same as that of the better-known PDCoV HKU15-155. The genome consists of a 5′ untranslated region (UTR), open reading frame 1a/1b (ORF1a/1b); the genes encoding the proteins spike (S), envelope (E), membrane (M), and nonstructural protein 6 (NS 6), nucleocapsid (N), and nonstructural protein 7 (NS 7); and a 3′ UTR.

Analysis of PDCoV SD with the basic local alignment search tool (BLAST) demonstrated that strain SD shares nucleotide identity percentages of 98.97%, 99.06%, and 99.13% with HKU15-155, CH/Sichuan/S27/2012, and CH-HB-2014, respectively (8). Moreover, this analysis revealed a 6-nt (TTTGAA) deletion at positions 1739 to 1744 (corresponding to the HKU15-44 sequence) in the Nsp-2 region and a 9-nt (TCGGCAATG) deletion at positions 2810 to 2818 in the Nsp-3 region.

The data presented here will facilitate future research on the epidemiology and diagnosis of disease caused by PDCoV infection in China. Further study remains to be conducted to develop effective measures to control this disease.

### Accession number(s).

The genome sequence of the porcine deltacoronavirus strain SD was deposited in GenBank with the accession no. MF431743.
